# Association of the rs7216389 Polymorphism in Orosomucoid-Like 3 (ORMDL3) Gene With Childhood Asthma: A Multicenter Case-Control Study

**DOI:** 10.7759/cureus.104571

**Published:** 2026-03-02

**Authors:** Qudsia U Khan, Maheen Nasir, Aimen B Asif, Junaid A Khan, Kanzul Eman, Muhammad Farid

**Affiliations:** 1 Physiology, CMH Lahore Medical College and Institute of Dentistry, Lahore, PAK; 2 Internal Medicine, Capital Development Authority Hospital, Islamabad, PAK; 3 Medicine, CMH Lahore Medical College and Institute of Dentistry, Lahore, PAK; 4 Opthalmology, CMH Lahore Medical College and Institute of Dentistry, Lahore, PAK; 5 Internal Medicine, CMH Lahore Medical College and Institute of Dentistry, Lahore, PAK; 6 Psychiatry and Behavioral Sciences, CMH Lahore Medical College and Institute of Dentistry, Lahore, PAK

**Keywords:** associated factors of asthma, genetics, pediatric asthma, polymorphism, rs7216389 polymorphism in orosomucoid-like 3 gene

## Abstract

Background

Asthma is recognized worldwide as the most common chronic illness in children, and numerous environmental and genetic factors have been identified in its pathophysiology. Among genetic contributors, the orosomucoid-like 3 (ORMDL3) gene, a negative regulator of sphingolipid synthesis, has gained particular importance and has been significantly linked to the onset of asthma. This proposed connection highlighted the importance of comprehensive in vivo analysis and exploration of the effect of ORMDL3 on asthma immunopathology. The present study aimed to analyze and compare the distribution pattern of the ORMDL3 gene among children with and without asthma in order to evaluate its association with childhood asthma susceptibility.

Methods

This multicenter study, with a case-control design, was conducted across three institutes, namely, CMH Lahore Medical and Dental College, the University of Health Sciences, and the Children's Hospital Lahore, from March 3, 2021, to May 21, 2022. In this study, 50 asthmatic and 50 non-asthmatic children were enrolled, with participants ranging in age from three to 18 years and having a confirmed diagnosis of genetic asthma. For analysis of genotypic distribution and associations, the genomic DNA was first extracted and quantified using gel electrophoresis. Single-nucleotide polymorphism (SNP) genotyping was then performed using the tetra-primer amplification refractory mutation system-polymerase chain reaction (ARMS-PCR). Genotypic associations were then analyzed, with focus on rs7216389, with the C-allele considered more common in healthy individuals and the T-allele more frequent in asthmatic children.

Results

With respect to the primary objective, analysis of the ORMDL3 rs7216389 polymorphism in our study found the heterozygous T/C genotype to be the most frequent among the asthmatic patients (n=34; 68%), and there was no significant observable difference between the genotype of asthmatic and non-asthmatic children. Altogether, a statistically significant association was not identified between the rs7216389 polymorphism and asthma status. In addition, seasonal fluctuations significantly (p<0.001) influenced asthma symptoms, with the majority proportion of asthmatic patients (n=30; 60%) reporting an exacerbation of symptoms with seasonal changes. Similarly, asthma symptoms were significantly exacerbated by animal and dust allergens, with p-values of 0.010 and 0.018, respectively.

Conclusion

In conclusion, no association was found between the ORMDL3 rs7216389 polymorphism and childhood asthma in our study. These findings suggest that this variant is unlikely to serve as an independent predictor of asthma in children. Future research with a larger sample size, a diverse population, and a wider genomic analysis is recommended to better understand the genetic architecture of childhood asthma and to determine whether an association exists.

## Introduction

Asthma is a chronic inflammatory disease of the respiratory tract, with one in 11 children currently receiving treatment. It remains the most common chronic disease of childhood, although there has been a recently observed decline in the number of newly diagnosed pediatric cases [1]. Asthma can be broadly classified into atopic and non-atopic types. Classically defined, non-atopic asthma refers to chronic inflammation and obstruction of the conducting airways driven by intrinsic factors, whereas atopic asthma describes a disease phenotype induced by type 1 hypersensitivity reactions to antigens of common environmental factors [2].

Globally, asthma accounts for around 180,000 deaths each year [3]. More than 80% of these fatalities occur in low- and middle-income nations, where over 70% of these countries are unable to provide their residents with an appropriate supply of World Health Organization (WHO)-designated critical asthma drugs [4].

Chest tightness, dyspnea, wheezing, and coughing are all hallmark symptoms of asthma. Asthma severity is typically classified into intermittent, mild, moderate, and severe categories based on the frequency of symptoms, response to therapy, and the existence of symptoms between exacerbations. Of these, severe asthma is of particular concern for the researchers and healthcare professionals, as it carries the highest risk of hospitalization and mortality [5].

The diagnosis of asthma can be challenging and is based on recognizing the characteristic respiratory symptoms, as well as the existence of fluctuating airflow restriction. Diagnosis is more likely if symptoms occur at night, after activity, or after exposure to common allergens [6]. Risk factors frequently implicated in pediatric asthma include a family history of asthma and either a personal or family history of atopic conditions such as allergic rhinitis or eczema. The standard diagnostic approach involves an assessment of symptoms and risk factors combined with spirometry, particularly the measurement of forced expiratory volume in one second (FEV1). Greater variance in lung function measured over time poses a greater risk of asthma. The diagnosis can be further strengthened with bronchial provocation tests using methacholine or exercise [7].

As there is no definitive cure for asthma, a physician's primary aim is either to eliminate symptoms or to decrease the severity and number of episodes. A stepwise approach, using both pharmacological and non-pharmacological interventions, can be used for management [8]. Non-pharmacological therapy includes patient education, which enables them to understand the nature of the disease, helps them to identify triggers, and provides strategies to prevent exacerbations. Pharmacological interventions include bronchodilators, primarily beta-2 agonists, which provide a rapid relief of symptoms, and anti-inflammatory medications such as corticosteroids, which prevent the delayed inflammatory effects of asthma [9]. 

In the development of childhood asthma, multiple environmental factors have been identified and studied in depth; however, another significant factor that may contribute to the onset of the disease remains widely understudied, and that is the individual's own genetic makeup [10]. A genome-wide association study conducted in 2007 examined single-nucleotide polymorphisms (SNPs) among 994 children with asthma and 1234 healthy controls and made a groundbreaking revelation of the presence of a strong association between childhood asthma and the SNPs located near the 17q21 genetic locus [11]. Although the locus contains several genes, the strongest association with pediatric asthma was observed in the SNP-driven overexpression of the orosomucoid-like 3 (ORMDL3) gene, a negative regulator of sphingolipid synthesis, in lymphoblastoid cell lines transformed by the Epstein-Barr virus (EBV). Among the variants identified, the SNP most closely associated with the onset of asthma was rs7216389. This polymorphism is marked by the presence of a cytosine allele (C) in non-asthmatics and a higher frequency of the thymine (T) allele in asthmatics [12]. The revelation of a significant connection of the development of asthma with ORMDL3 identified a need for comprehensive in vivo analysis and exploration of the underlying mechanisms. It has been demonstrated that the ORMDL3 gene expression is induced in various cell types on exposure to an allergen, and this overexpression leads to an increase in levels of genes that encode pro-inflammatory cytokines, such as IL-8, CXCL-10, CXCL-11, and CCL-20, indicating a potential immunopathological pathway that connects this gene to asthma development [13]. 

Given these findings, the present study aimed to compare the pattern of rs7216389 polymorphisms of the ORMDL3 gene among children with and without asthma and to determine whether there is an association of the variant with the development of childhood asthma.

## Materials and methods

This multicenter study, with a case-control design, was conducted in accordance with the clinical practice guidelines across three institutions of Lahore, namely, CMH Lahore Medical and Dental College, the University of Health Sciences (UHS), and the Children's Hospital [14-16], after obtaining approval from the Institutional Review Board of CMH Lahore Medical College and Institute of Dentistry (approval number: CASE#.536/ERC/CMH/LMC). The study duration was 15 months, from March 3, 2021, to May 21, 2022. Cases and controls were frequency-matched by age.

Sampling and sample size

A probability purposive sampling technique was employed. Using the finite population correlation formula $\mathrm{n}=\mathrm{Z}^{2}\times\mathrm{p}\left(1&minus;\mathrm{p}\right)/\mathrm{d}^{2}$, a sample size of 100 participants was calculated, ensuring a 95% confidence interval and a 5% absolute precision. The cohort was divided equally into 50 non-asthmatics and 50 asthmatics. Written informed consent was taken from the children's parents or guardians prior to the collection of data. The inclusion and exclusion criteria for the study participants are summarized in Table 1 [17]. Asthma diagnosis was based on documented clinical evaluation and pulmonary function testing at the time of diagnosis according to the Global Initiative for Asthma (GINA) 2019 guidelines. Pulmonary function tests (PFTs) were not performed as part of this study protocol.

**Table 1:** Inclusion and exclusion criteria for the study participants GINA: Global Initiative for Asthma

Group	Inclusion criteria	Exclusion criteria
Asthmatic participants	Age 3-18 years	Acute lung infection
	Confirmed diagnosis of asthma according to GINA 2019 guidelines	
	Presence of asthma-related symptoms (cough, wheeze, breathlessness)	Congenital lung diseases (e.g., cystic fibrosis, congenital emphysema)
	Imaging confirming no other underlying lung disease	
Non-asthmatic participants	Age 3-18 years	Any asthma or asthma-like symptoms
	No history of diagnosis or treatment of asthma	
	Absence of asthma-like symptoms and allergies	

Blood sampling

A 2 ml peripheral blood sample was collected from each patient and moved to ethylenediaminetetraacetic acid (EDTA)-anticoagulated tubes. Before processing, the stored samples were kept at 4°C. Vacutainer tubes were transferred to the Human Genetics and Molecular Biology Department of UHS, Lahore, for analysis. 

DNA extraction and quantification

Genomic DNA was extracted from blood samples by using a commercially available kit (Zymo Research, Irvine, California, United States) by following the manufacturer's protocol. The DNA quantity and purity were estimated using spectrophotometry (NanoDrop technology, Thermo Fisher Scientific, Waltham, Massachusetts, United States).

Genotyping 

The beta-2 microglobulin (B2M) nuclear DNA sequence and the mitochondrial tRNA-Leu (UUR) sequence were obtained from the National Center for Biotechnology Information (NCBI) GenBank. Using the University of California, Santa Cruz (UCSC) Genome Browser, in silico polymerase chain reaction (PCR) was performed using primers created with Primer 3. Primers were optimized by gradient PCR using a temperature of 58-64°C and amplified using real-time quantitative PCR. The tetra-primer amplification refractory mutation system (ARMS)-PCR was used for genotyping. PCR product specificity was verified using agarose gel electrophoresis, carried out at 120 volts for 30-40 minutes, followed by gel visualization and sequencing. Genotype distribution in the control group was assessed for the Hardy-Weinberg equilibrium using the chi-squared test.

Statistical analysis

Normality of continuous variables was assessed using the Shapiro-Wilk test. As the primary variables were categorical, associations were evaluated using the chi-squared test. Odds ratios (ORs) with 95% confidence intervals (CIs) were calculated to estimate the strength of association. Degrees of freedom and p-values were reported, with p<0.05 considered statistically significant. No multivariable adjustment for potential confounders was performed. Statistical analyses were conducted using IBM SPSS Statistics for Windows, Version 26.0 (IBM Corp., Armonk, New York, United States).

## Results

Among both groups, the distribution according to gender comprised 22 females (44%) and 28 males (56%) in the non-asthmatic group and 20 females (40%) and 30 males (60%) in the asthmatic group, for a total of 50 participants in each group. Table 2 summarizes the distribution of participants according to age in both groups. The majority of asthmatic participants were aged >12 years (n=16; 32%), while the highest proportion of non-asthmatic participants belonged to the 3-5-year age group (n=16; 32%). No statistically significant difference was observed in age distribution between the two groups. 

**Table 2:** Distribution of patients according to age

Group	Age group (years)	Frequency (n)
Asthmatic participants	3-5	15 (30%)
	6-8	8 (16%)
	9-11	11 (22%)
	>12	16 (32%)
	Total	50 (100%)
Non-asthmatic participants	3-5	16 (32%)
	6-8	14 (28%)
	9-11	15 (30%)
	>12	5 (10%)
	Total	50 (100%)

**Figure 1:**
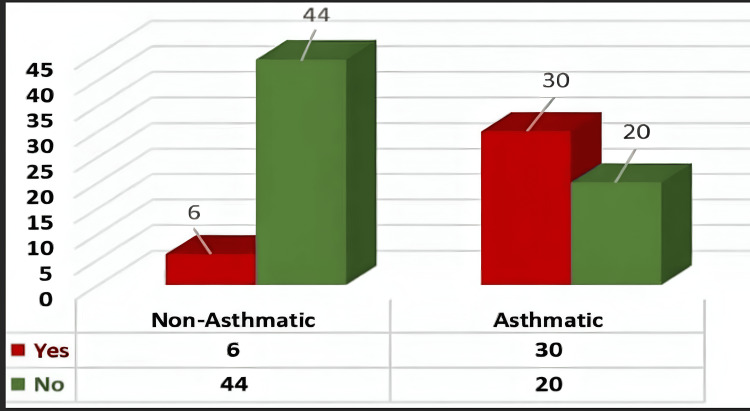
Distribution of any seasonal influence over asthma symptoms in patients

Analysis of the data revealed that seasonal changes had a highly significant influence over the symptoms of asthma (p<0.001). Among individuals with asthma, 30 out of 50 (60%) reported an exacerbation of symptoms with seasonal changes, as shown in Figure 1.

The results were analyzed for environmental factors that may potentially influence the symptoms of asthma in patients. It was found that animal allergy and dust allergy significantly aggravated the symptoms in patients, with statistically significant p-values of 0.010 and 0.018, respectively. The degree of freedom was 3, and the effect size was 2, so there was a moderate association.

**Figure 2:**
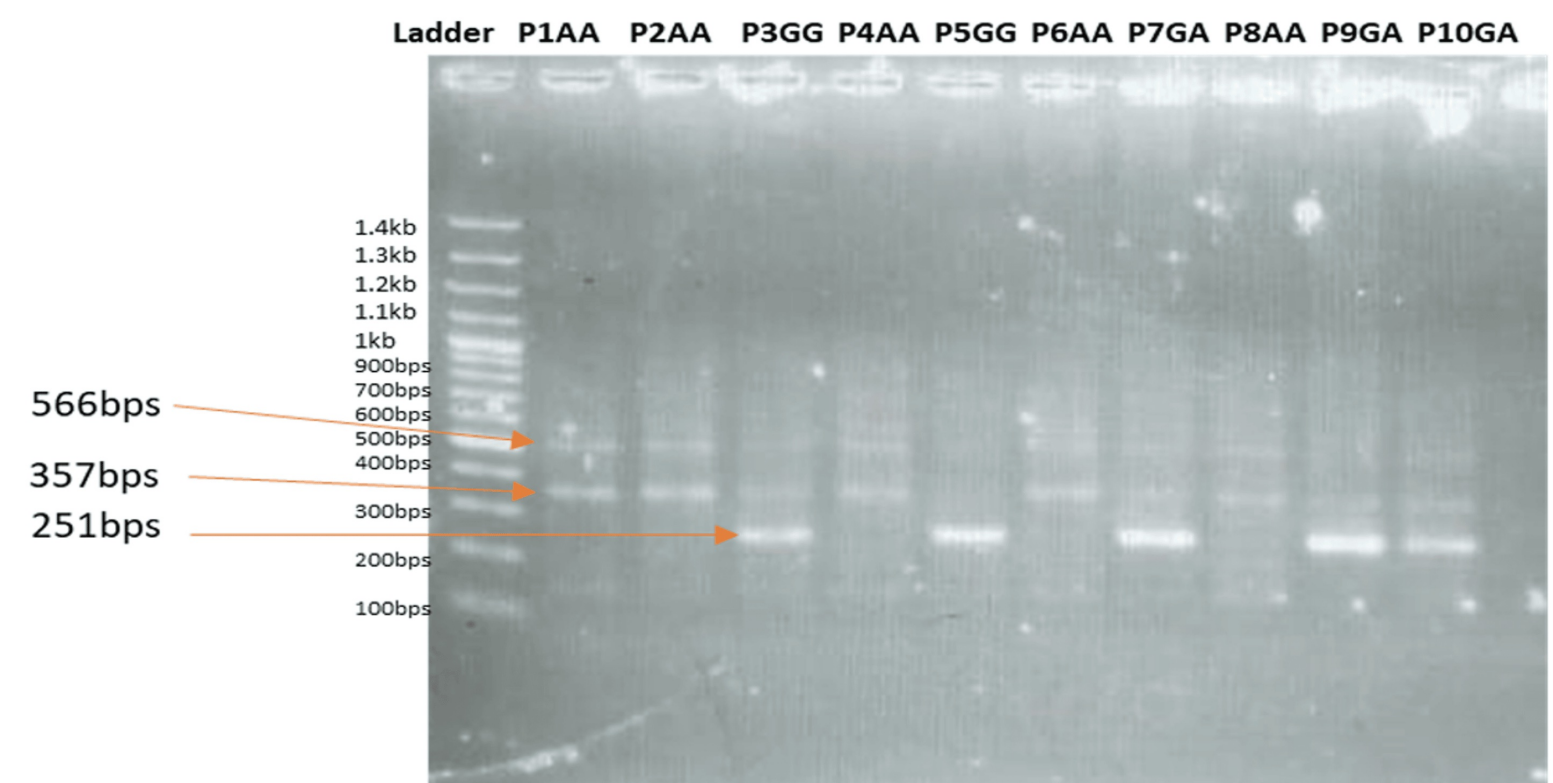
Genotyping result of SNP rs7216389 in the ORMDL3 gene of six patients A band of 566 bps: control band. Two bands of 566 bps and 357 bps: T/T genotype. Two bands of 566 bps and 251 bps: C/C genotype. Three bands of 566 bps, 357 bps, and 251 bps: T/C genotype. T/T: wild-type homozygous; C/C: homozygous mutant; T/C: heterozygous mutant; SNP: single-nucleotide polymorphism; ORMDL3: orosomucoid-like 3

Genotypic distribution was analyzed and compared across the two groups to assess the distribution pattern. In the homozygous (C/C) genotype, two bands, i.e., a control band and the corresponding band, appeared, whereas in the case of heterozygosity (T/C), three bands appeared, as illustrated in Figure 2. 

The genotypic distribution revealed that the heterozygous T/C genotype was the most prevalent among the asthmatic patients (n=35; 70%), followed by the wild-type T/T genotype (n=10; 20%) and the rare homozygous C/C genotype (n=5; 10%), whereas, in the control group, the percentage frequency of T/T, T/C, and C/C genotypes was 40.5% (n=20), 52.4% (n=26), and 7.1%(n=4), respectively. 

Logistic regression was used to obtain ORs in the presence of more than one explanatory variable while keeping the T/T genotype as the reference category. The wild-type homozygous (T/T) variable was observed in 40.5% (n=20) of non-asthmatics and 20% (n=10) of asthmatics. The heterozygous mutant (T/C) type variant was 52.4% (n=24) in the control group and 70% (n=35) in the asthmatic group, revealing an OR of 2.53 (95% CI: 0.95-6.71; p=0.084), indicating an insignificant association, as also depicted by the degree of freedom=2 and effect size=0.22. The homozygous mutant (C/C) genotype was present in 10% (n=5) of asthmatics and only in 7.1% (n=4) of non-asthmatics, with an OR of 2.37 (95% CI: 0.43-13.24; p=0.342). Overall, the genotypic distribution across the two study groups showed no significant difference and showed no association with the disease, as shown in Table 3.

**Table 3:** Genotype distribution and the odds ratios of SNP rs7216389 in the cases and healthy controls *p>0.05 not significant T/T: wild-type homozygous; C/C: homozygous mutant; T/C: heterozygous mutant; SNP: single-nucleotide polymorphism

Genotype (rs7216389)	Controls, n (%)	Cases, n (%)	Odds ratio (95% CI)	P-value
T/T	20 (40.5%)	10 (20%)	1.00	-
T/C	26 (52.4%)	35 (70%)	2.53 (0.95-6.71)	0.084*
C/C	4 (7.1%)	5 (10%)	2.37 (0.43-13.24)	0.342

## Discussion

This study was conducted with the objective of investigating the association of the rs7216389 polymorphism in the ORMDL3 gene with childhood asthma, and genotypic analysis in our cohort revealed no significant association. In contrast, a recent study conducted in the United States in 2020 reported a significant relationship between rs7216389 and ORMDL3 overexpression, which in turn correlated with increased severity of asthma. Children from two different regions of the United States were recruited, and several asthma-associated 17q21 genotypes (rs7216389, rs8076131, rs4065275, rs12603332, and rs8067378) were analyzed, and a strong association with asthma susceptibility was found [18].

In 2018, a study was done in Pakistan to investigate the association of rs7216389 with the susceptibility of asthma by analyzing 50 asthmatic cases and 50 healthy controls recruited from Islamabad and Lahore. The researchers selected 16 SNPs from 10 candidate genes and, after genotyping, found the minor allele frequency to be 0.413, which is comparable to the frequency observed in the present study. Despite this, no significant association was found between rs7216389 and asthma susceptibility [19]. These findings from the same region, yielding similar results, reinforce our findings of non-association of rs7216389 with childhood asthma.

A Jordanian study conducted in 2016 further supports the findings of our study. They recruited participants in two groups: adults and children. Genotyping revealed that the heterozygous genotype T/C was significantly associated with childhood asthma, whereas no significant association was discernible in adult asthmatic patients [20].

Yang and colleagues investigated the genetic association of SNPs in and around the ORMDL3 gene with childhood asthma in a Chinese population. The study included 152 Chinese asthmatic children, diagnosed according to GINA guidelines, and 190 inpatient controls who had non-asthmatic conditions and no history of asthma. Six tag SNPs in and around the ORMDL3 gene were genotyped, and rs7216389 was found to be significantly associated with asthma in children. The genotypic frequency patterns in their cohort differed from those observed in the present study: the wild-type T/T genotype was found to be the most prevalent, followed by heterozygous T/C and mutant C/C genotypes [21]. Earlier studies in European populations also reported the same association pattern and found rs7216389 to be contributing to childhood asthma susceptibility [22]. These discrepancies compared to our findings may be explained by differences in sample size and genetic background, as the genetic makeup of the Chinese and European populations differs substantially from that of populations in Pakistan.

There are a number of potential explanations for why ORMDL3 deletion did not influence house dust mite (HDM)-induced allergic airway disease parameters. First, it is possible that a general regulatory function of ORMDL3, affecting allergen-induced immunopathology, is only induced upon gene overexpression, as the two other members of the ORMDL protein family, ORMDL1 and ORMDL2, might compensate for the loss [23]. All three proteins have been reported to show a high level of amino acid homology, and in vitro, only the cumulative knockdown of the whole gene family is known to induce a significant increase in ceramide levels, which suggests that they share the same regulatory activity on sphingolipid synthesis and that compensatory effects are to be expected, which can be further explained by time-course experiments aimed at elucidating the gene expression patterns of all ORMDL genes following allergen challenge [24].

Additionally, ORMDL3 could have been misidentified in encoding a potential regulator of asthma onset, and another gene or altered gene expression of multiple genes throughout the 17q21 locus could account for the increased susceptibility. Evidence, however, for ORMDL3 being associated with asthma onset is very strong, as the linkage has been verified in multiple reports [25]. Nonetheless, it has been reported that SNPs showing the highest association between ORMDL3 upregulation and asthma disease are not located within the protein coding sequence of ORMDL3 or in close proximity to the transcription promoter region, but are in fact further upstream in the first intron of the neighboring gene GSDMB [26,27]. This, of course, does not exclude that these SNPs are located in a genetic unit comprising regulatory activity on ORMDL3 gene expression, but equally they might additionally regulate other genes inside the 17q21 locus [28-30].

There are several limitations to this study, which ought to be considered when interpreting the findings. The statistical power to detect modest genetic associations between the ORMDL3 rs7216389 polymorphism and childhood asthma may have been limited by the relatively small sample size. Additionally, although the present study was a multicenter study, participants were recruited from the same geographical area, which may limit the generalizability of the results to other populations with different genetic backgrounds and environmental influences. Finally, as it was a case-control study, causal inferences cannot be established, and to better evaluate the temporal relationship between genetic variants, environmental triggers, and asthma development, longitudinal studies are required. 

## Conclusions

In conclusion, our findings indicate that the ORMDL3 rs7216389 polymorphism does not demonstrate a significant association with childhood asthma in this cohort and, at least in this population, is unlikely to serve as an independent predictor of childhood asthma. Seasonal changes had a highly significant influence on the symptoms of asthma. Most of the asthmatic patients experienced asthma-related symptoms when the season changed. Interestingly, the heterozygous T/C genotype was the most abundant, and the percentage was significantly high in asthmatic patients, but the association with childhood asthma and heterozygous T/C was not established, as the p-value was more than ≥0.05. Nevertheless, asthma is a multifactorial disease influenced by complex interactions between environmental and genetic factors; therefore, further studies incorporating larger sample sizes, greater geographical diversity, and more comprehensive genomic analysis are warranted. Such efforts may provide deeper insights into the genetic basis of childhood asthma and help clarify whether a potential association with ORMDL3 or related pathways exists in broader populations. 
